# MP2RAGE multispectral voxel‐based morphometry in focal epilepsy

**DOI:** 10.1002/hbm.24756

**Published:** 2019-08-12

**Authors:** Raviteja Kotikalapudi, Pascal Martin, Michael Erb, Klaus Scheffler, Justus Marquetand, Benjamin Bender, Niels K. Focke

**Affiliations:** ^1^ Department of Diagnostic and Interventional Neuroradiology University Hospital Tübingen Tübingen Germany; ^2^ Department of Neurology and Epileptology, Hertie Institute for Clinical Brain Research University Hospital Tübingen Tübingen Germany; ^3^ Department of Clinical Neurophysiology University Hospital Göttingen Göttingen Germany; ^4^ Department of Biomedical Magnetic Resonance University Hospital Tübingen Tübingen Germany; ^5^ Max Planck Institute for Biological Cybernetics Tübingen Germany

**Keywords:** epilepsy, lesion detection, MP2RAGE, MRI‐negative, smoothing, statistical thresholds, VBM

## Abstract

We assessed the applicability of MP2RAGE for voxel‐based morphometry. To this end, we analyzed its brain tissue segmentation characteristics in healthy subjects and the potential for detecting focal epileptogenic lesions (previously visible and nonvisible). Automated results and expert visual interpretations were compared with conventional VBM variants (i.e., T1 and T1 + FLAIR). Thirty‐one healthy controls and 21 patients with focal epilepsy were recruited. 3D T1‐, T2‐FLAIR, and MP2RAGE images (consisting of INV1, INV2, and MP2 maps) were acquired on a 3T MRI. The effects of brain tissue segmentation and lesion detection rates were analyzed among single‐ and multispectral VBM variants. MP2‐single‐contrast gave better delineation of deep, subcortical nuclei but was prone to misclassification of dura/vessels as gray matter, even more than conventional‐T1. The addition of multispectral combinations (INV1, INV2, or FLAIR) could markedly reduce such misclassifications. MP2 + INV1 yielded generally clearer gray matter segmentation allowing better differentiation of white matter and neighboring gyri. Different models detected known lesions with a sensitivity between 60 and 100%. In non lesional cases, MP2 + INV1 was found to be best with a concordant rate of 37.5%, specificity of 51.6% and concordant to discordant ratio of 0.60. In summary, we show that multispectral MP2RAGE VBM (e.g., MP2 + INV1, MP2 + INV2) can improve brain tissue segmentation and lesion detection in epilepsy.

## 1 INTRODUCTION

Focal epilepsy constitutes ~60% of all epilepsies (Rosenow & Luders, 2001), and is characterized by “focal onset,” that is, seizures originating in a local brain region (Scheffer et al., 2017). About 30% of focal epilepsy patients experience disabling seizures medically refractory/resistant to anticonvulsants (Kwan & Brodie, 2000). For these patients, epilepsy surgery can be very beneficial, provided a resectable, focal area of seizure onset is identifiable (Wiebe et al., 2001). Before surgery in these patients, a presurgical evaluation is conducted, which includes brain MRI as an important modality. Cortical malformations, mainly focal cortical dysplasias (FCD), are one of the common causes associated with refractory focal epilepsy that are identifiable via an MRI. Typical imaging features are blurred gray–white matter (GM–WM) junctions, cortical thinning/thickening, and hypo‐ or hyperintense MR signal (Blumcke et al., 2011). However, a relevant proportion of lesions escape visual detection. Approximately half (30–50%) of patients undergoing surgery without MRI visible lesion eventually have cortical dysplasia/FCD upon histological investigations (Bernasconi, Bernasconi, Bernhardt, & Schrader, 2011; Wang et al., 2013). Potential reasons for missing an FCD are their subtlety and, at times their small size as well as variable location. Scans with missed FCDs are mostly considered as normal MRI (MRI‐negative patients). Consequently, patients have worse surgical outcomes compared to cases with visible lesions on MRI (Tellez‐Zenteno, Hernandez Ronquillo, Moien‐Afshari, & Wiebe, 2010). Technical advances of the static magnetic fields from 1.5 to 3 T and higher field strengths of 7 T can aid in detecting epileptogenic lesions that are otherwise difficult to perceive (De Ciantis et al., 2016; Knake et al., 2005; Veersema et al., 2017; Zijlmans et al., 2009). Also, computational methods have been shown helpful in detecting subtle lesions, previously missed in the visual analysis (Kotikalapudi et al., 2018; Martin, Bender, & Focke, 2015; Martin et al., 2017). Voxel‐based morphometry (VBM) is a well‐established method for computer‐aided, volumetric MRI processing (Ashburner & Friston, 2000). Usually applied on T1 images (T1 VBM), it can successfully detect local GM concentration/volume changes in patients with a known lesion in focal epilepsy (Bonilha et al., 2006; Colliot et al., 2006; Martin et al., 2015). VBM is based on tissue segmentation, where the brain is classified into three main tissue categories, namely: GM, WM, and cerebrospinal fluid (CSF) (Ashburner & Friston, 2005). MRI technical factors (e.g., image bias) directly impact on the quality of tissue segmentation and influence the VBM process (Focke et al., 2011). Improved tissue segmentation can be achieved through combining multispectral MRI contrasts in the segmentation process (Alfano et al., 1997; Ashburner & Friston, 1997; Ashburner & Friston, 2005; Fletcher, Barsotti, & Hornak, 1993; Lambert, Lutti, Helms, Frackowiak, & Ashburner, 2013; Vannier et al., 1985). Recent studies have confirmed superiority of multispectral over conventional T1‐only segmentation by addressing issues such as overestimation of dura and vessels as GM and also improve cortical GM segmentation (Lindig et al., 2018; Viviani, Pracht, et al., 2017; Viviani, Stocker, et al., 2017). With the addition of T2/T2‐FLAIR weighted, VBM variants (T1 + FLAIR, T1 + T2) have also shown improved lesion detection over T1‐only VBM, in both lesional and non lesional MRI focal epilepsy (Kotikalapudi et al., 2018; Lindig et al., 2018).

Particularly at higher field strengths of ≥3 T, image bias due to static magnetic (B0) and radio‐frequency field (transmission B1^+^ and reception B1^−^) inhomogeneities are problematic for segmentation algorithms (Focke et al., 2011). One option to improve signal intensity inhomogeneities has been described by primarily acquiring 2 MPRAGE (hence MP2RAGE) images with different inversion times, otherwise keeping sequence parameters identical (Marques et al., 2010; Van de Moortele et al., 2009). The resultant images show enhanced contrast‐to‐noise ratio (especially GM–WM contrast), independent of *T*_2_*, proton density, B1^−^, and reduced B1^+^ inhomogeneities. Thus, acquired images are, at least partially, corrected for image bias intrinsically and have been called “self‐bias corrected images.” Newer studies have shown promise of MP2RAGE in improving visualization of lesions (Beck et al., 2018; Pittau et al., 2018). Moreover, reduced intensity inhomogenities should also improve tissue segmentation, which facilitates VBM analysis for lesion detection (Ashburner & Friston, 2005). However, MP2RAGE and the multispectral MP2RAGE variants have not been systematically analyzed for detecting subtle epileptogenic lesions in a VBM approach. Moreover, it was previously shown that the performance of lesion detection is strongly influenced by the choice of smoothing and statistical thresholds or *t*‐scores (Kotikalapudi et al., 2018; Martin et al., 2017). The ideal parameters for an MP2RAGE VBM are yet unclear in focal epilepsy.

Hence, the major aim of this study is to assess MP2RAGE‐VBM (e.g., MP2 + INV1, MP2 + INV2, MP2‐alone) in focal epilepsy patients. To this end, we first assessed systematic differences of MP2RAGE versus T1 and T1 + FLAIR multispectral tissue segmentation in healthy controls. Subsequently, the diagnostic performance of MP2RAGE‐VBM in focal epilepsy with and without visible lesions (MRI‐negative) was quantified using a lobar hypothesis. These results will offer guidance in applications of MP2RAGE‐VBM (and its multispectral combinations) in contrast to conventional T1 and T1 + FLAIR VBM.

## 2 METHODS

### 2.1 Data acquisition

We recruited 31 healthy controls (14 females, mean age = 28.4) and 21 patients (7 females, mean age = 31.5) with focal epilepsy. Five of the focal epilepsy patients had a visible lesion concordant with malformation of cortical development (MCD). Sixteen patients were labeled as MRI‐negative on expert neuroradiology review. All patients had undergone comprehensive presurgical diagnostics including noninvasive video‐EEG telemetry, neuropsychological assessment (except case ID 11) and epilepsy‐dedicated 3 T‐MRI. Six patients have undergone invasive EEG and three patients have been surgically resected with histology showing evidence for FCD Type II b in two patients. Clinical details of patients are summarized in Data S1—Table S1. A clinical hypothesis of epilepsy onset on a lobar level was derived through expert consensus in the epilepsy case conference with all available information in the presurgical epilepsy program. VBM findings analyzed in this study were not considered for forming the clinical hypothesis.

Imaging data was acquired on 3 T Siemens Magnetom Prisma MRI at the University Hospital Tübingen, Germany. The acquisition protocol consisted of 3D T1‐weighted Magnetization‐Prepared Rapid Gradient‐Echo (MPRAGE) [TE = 2.98 ms, TR = 2,300 ms, TI = 900, flip angle = 9°, acquisition time = 5:12 min:s], a 3D T2‐weighted Sampling Perfection with Application optimized Contrasts using different flip angle Evolution‐Fluid‐Attenuated Inversion Recovery (T2‐SPACE FLAIR) [TE = 388 ms, TR = 5,000 ms, TI = 1800 ms, flip angle = 120°, acquisition time = 6:32] and a 3D T1‐weighted Magnetization‐Prepared 2 Rapid Gradient‐Echo (MP2RAGE) [INV1 or TI1 = 700 ms, flip angle = 4° and INV2 or TI2 = 2,500 ms, flip angle = 5°, TE = 2.98 ms, TR = 5,000 ms, acquisition time = 8:52] using a 64 channel head coil with an isotropic resolution of 1 mm^3^. Subjects were considered for image postprocessing steps only when T1, FLAIR, and MP2RAGE images were available and did not have relevant artifacts (motion in particular) in the visual quality control. All datasets/subjects passed this condition.

### 2.2 Image processing

All DICOM images were converted to NIFTI file format using mriconvert (http://www.lcni.uoregon.edu/~jolinda/MRIConvert). SPM12 (http://www.fil.ion.ucl.ac.uk) based on MATLAB R2016a (The Math Works, Natick, MA) was used for image processing. MP2 images were reconstructed using the manufacturer's software, which uses the following equation for reconstruction:

$$\mathrm{MP}2=\frac{\mathrm{INV}1^{*}\;\mathrm{INV}2}{{\left|\mathrm{INV}1\right|}^{2}+{\left|\mathrm{INV}2\right|}^{2}}$$

Detailed mathematical calculations and equations for MP2 image reconstruction are available in the original studies (Marques et al., 2010). VBM was performed for single‐contrast T1, MP2, and multispectral combinations, namely: T1 + FLAIR, MP2 + FLAIR, MP2 + INV1, MP2 + INV2, and INV1 + INV2. For multispectral combinations, linear co‐registration was performed using a normalized mutual cost function with 12° of freedom. For co‐registration, reference images were T1 (in T1 + FLAIR), MP2 (in MP2 + FLAIR, MP2 + INV1, and MP2 + INV2), and INV1 (in INV1 + INV2), whereas the latter images in the combinations served as the source images. Unified segmentation (Ashburner & Friston, 2005) was applied with default settings of bias regularization 0.0001 and bias cutoff FWHM 60 mm. Next, GM, WM, and CSF tissue probability maps were spatially normalized with an isotropic resolution of 1mm^3^ using Diffeomorphic Anatomical Registration Through Exponentiated Lie Algebra (DARTEL), based on the respective native space GM and WM maps and building a custom template in MNI space for each multispectral combination (Ashburner, 2007). During the normalization process, images were modulated to preserve tissue quantity (in case of group level analysis) and unmodulated images to preserve tissue concentration (in case of individual subject vs. group analysis) (Good et al., 2001). As a final step, images were smoothed using Gaussian kernel sizes 4–16 mm (step size of 2 mm) full width at half maximum (FWHM).

### 2.3 Comparisons for absolute tissue volumes

Native segmented GM, WM, and CSF were used to calculate the tissue volumes for healthy controls across segmentation models. For this purpose, voxel values (ranging from 0 to 1) were summed and multiplied with the voxel volume (1 mm^3^) to yield a tissue class specific volume. To estimate the total intracranial volume (TIV), the absolute volumes of GM, WM, and CSF were added. To assess significant differences in segmentation models, one‐way repeated measures ANOVA was conducted in SPSS (IBM SPSS Statistics 22) with adjustment for multiple comparisons (Bonferroni).

### 2.4 Voxel‐based comparison of multispectral variants

Smooth normalize modulated GM, WM, and CSF images of healthy controls were used to perform group comparisons across different segmentation models. T1 + FLAIR segmentation was used as a reference, since this had the best overall segmentation quality in our previous work (Lindig et al., 2018). Each multispectral combination was then compared against T1 + FLAIR using a paired *t*‐test in SPM, at 4 mm smoothing and a statistical threshold of *p* < .05 FWE (family wise error). In the individual analysis, we compared each patient against all controls (patient comparison) and each control against the rest of the controls (after removing the control in question that is, control comparison) in SPM12 using statistical cutoffs from 2.5 to 6 (step size of 0.1). This analysis was repeated for all smoothing levels (4–16 mm in step size of 2).

### 2.5 Automated individual lobar analysis

We used the MNI structural lobar atlas provided with FSL version 5.0 (Collins, Holmes, Peters, & Evans, 1995; Mazziotta et al., 2001) for the automated lobar analysis. The atlas comprised of bilateral mask for frontal, parietal, temporal and occipital lobes. We extracted lobar regions‐of‐interest from the atlas based on the clinical hypothesis (please refer hypothesis lobes from Data S1—Table S1), for each patient. These lobes were considered as “concordant lobes,” that is, concordant to the clinical hypothesis, while nonconcordant lobes (remaining lobes) were labeled “discordant lobes” individually for each patient. Since we do not expect controls to have epileptogenic findings, all lobes (bilateral: frontal, temporal, parietal, and occipital lobes) were defined as discordant lobes for all controls.

#### 2.5.1 Performance parameters

Concordant rate C_R_ (or discordant rate D_R_) was calculated for patients as the percentage ratio of number of patients with at least one‐third of voxels in a VBM cluster overlapping with concordant lobe (or discordant lobe) to the total number of patients;

$$C_{R}=\left(\frac{\text{Number of patients with concordant findings}}{\text{Total number of patients}}\right)\times 100$$

$$D_{R}=\left(\frac{\text{Number of patients with discordant findings}}{\text{Total number of patients}}\right)\times 100$$

Specificity was defined as the percentage ratio of controls with no VBM findings to total number of controls (findings with less than 1/3rd of voxels inside the brain were considered as no finding);

$$S_{P}=\left(\frac{\text{Number of controls with}\;\mathrm{no}\;\text{findings}}{\text{Total number of controls}}\right)\times 100$$

C_R_, D_R_, and S_P_ were calculated for each smoothing levels 4–16 mm, across statistical cutoffs 2.5–6, for all VBM models.

### 2.6 Estimating smoothing and T‐threshold

The method to estimate the diagnostically ideal smoothing and T‐threshold has already been explained in detail in our previous work (Kotikalapudi et al., 2018). In brief, a single‐channel VBM variant is considered as a reference, that is, MP2 in our case. Receiver operating characteristic (ROC) curves are plotted for each smoothing, using C_R_ (concordant rate) and 100‐S_P_ (specificity or 1‐false positive rate) values generated across statistical cut‐offs. For the MP2 VBM, the smoothing level yielding the highest AUC was then selected for the further analysis. At this reference smoothing, the optimal T‐threshold was determined by the intersection between C_R_ and S_P_. If multiple T‐thresholds fulfilled the criterion, the point with highest C_R_ was chosen. As such, the selected T‐threshold can be seen as a balanced trade‐off between C_R_ and S_P_. Euclidean distances in the ROC plots are calculated between the consequently obtained C_R_/S_P_ point to the ideal C_R_/S_P_ point (i.e., 100, 100) as an overall performance marker that integrate both C_R_ and S_P_. Thus, lower Euclidean distances signify a better diagnostic test performance. To further validate our choice of smoothing and statistical cut‐offs, we additionally used these parameters on visible epileptogenic lesions (suspected MCDs *n* = 5).

### 2.7 Visual interpretation

Visual cross‐verification of VBM findings was done by one certified neuroradiologist with more than 10 years of experience. For group level analysis, structural differences were visually interpreted on native space across individual subjects, to assess if the VBM differences were evident on the native tissue maps. For individual level analysis, at the estimated smoothing level and T‐threshold for each subject, the VBM findings across models were combined and inverse transformed to native space using the deformation utility in SPM12. These native combined VBM findings were smoothed with 1 mm Gaussian kernel and were provided to the reviewer for scoring overlayed on T1, FLAIR, and MP2 images, using in‐house software written in MATLAB. The reviewer was given prior information of the two groups, that is, patients and controls. For the patient group, the reviewer was blinded toward the clinical hypothesis and the VBM model providing the findings, but was aware if the scan was from a patient or control. The reviewer visually interpreted the patient VBM findings with respect to known MR‐morphological features of FCDs/MCDs (hyperintensity in T2, blurred gray/white matter boundary, focal increase/decrease of gray matter volume, ventricular tail sign, abnormal sulcal, or gyral pattern) and localization of the finding (in or adjacent to gray matter). For patients the reviewer gave scores from 1: visible and potentially epileptogenic, 2: nonvisible but potentially epileptogenic, 3: visible and likely nonepileptogenic, 4: unclear/nonvisible, or 5: artifact. Features that did not give suspicion of a relevant epileptogenic lesion included white matter lesions, vessels, or perivascular spaces. Findings clearly attributable to artifacts (e.g., due to movement, field inhomogeneities) were labeled as such. For controls, each finding was rated as either 1: visible and likely nonepileptogenic, 2: unclear/nonvisible or 3: artifact. The nonepileptogenic label was given when finding was visible on one/more structural images but was not likely related to epilepsy (e.g., perivascular spaces or microangiopathy). Unclear labels were used whenever the findings were not clearly visible to be confirmed as either epileptogenic, nonepileptogenic, or as an artifact.

## 3 RESULTS

### 3.1 Group level differences across segmentation models

**Figure 1:**
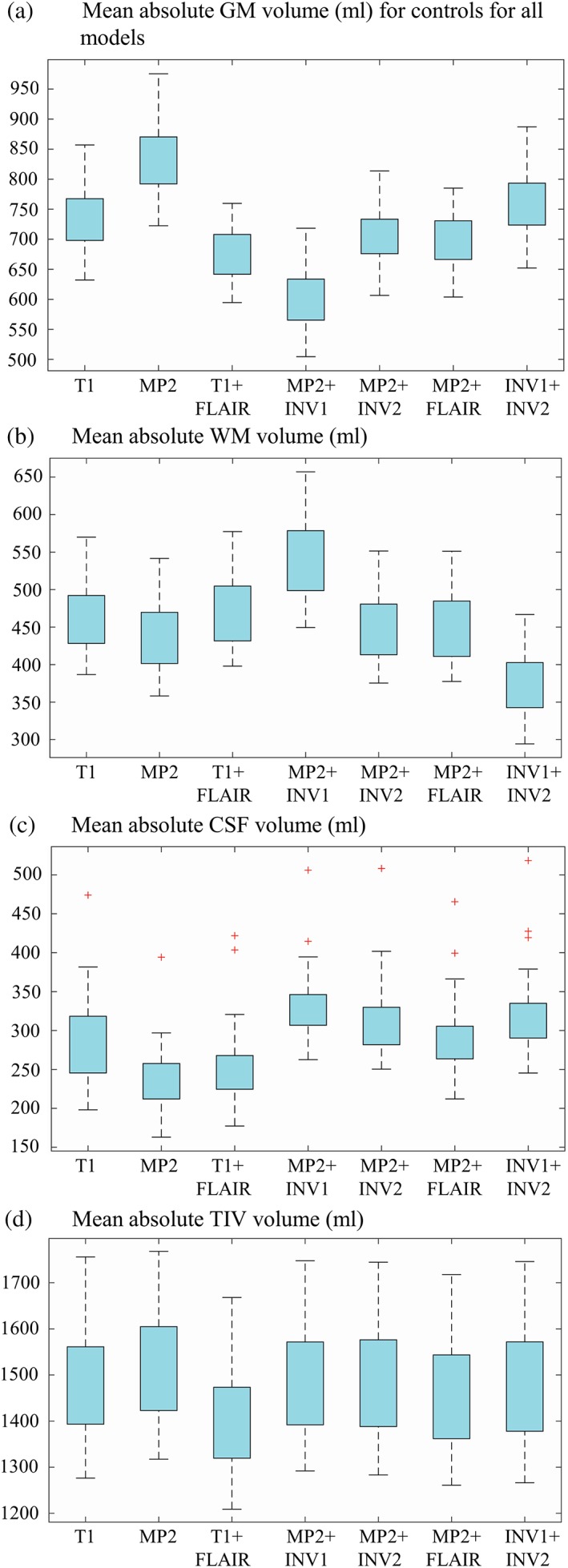
Mean of absolute volumes of gray matter (GM), white matter (WM), cerebrospinal fluid (CSF), and total intracranial volume (TIV) for controls across all models. A box plot representation of mean absolute volumes of GM (a), WM (b), CSF (c), and TIV (d) are present in this figure, across all VBM models [Color figure can be viewed at http://wileyonlinelibrary.com]

**Figure 2:**
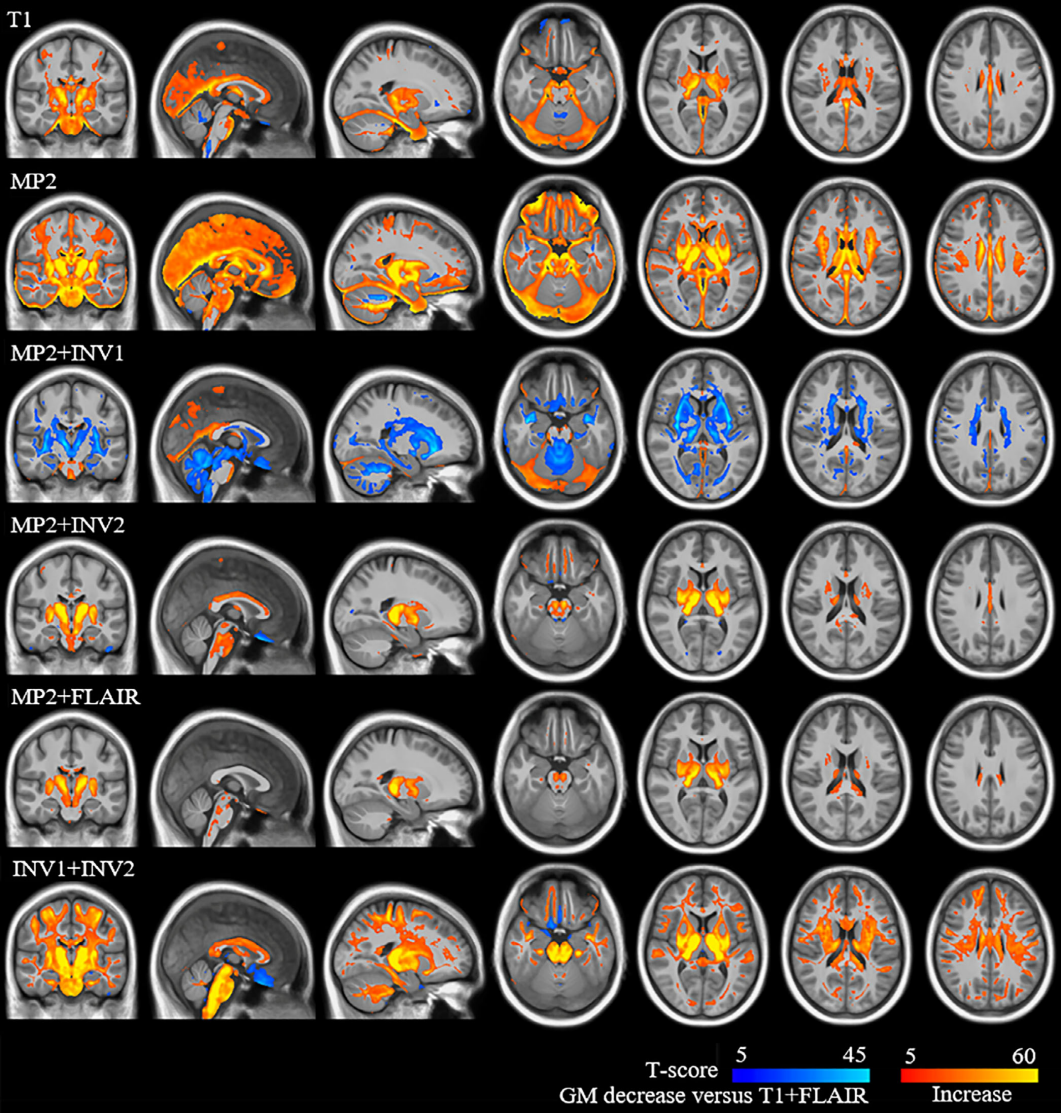
Group level differences for T1 + FLAIR with the rest of the models of GM increases and decreases for gray matter volume (modulated images) analysis. Group level comparison for T1 + FLAIR with the rest of the models is shown in this figure. The look‐up table with red‐yellow represents increased GM volumes in labeled models against T1 + FLAIR (models > T1 + FLAIR), while blue‐light blue represents decreased GM volumes (models > T1 + FLAIR) [Color figure can be viewed at http://wileyonlinelibrary.com]

**Figure 3:**
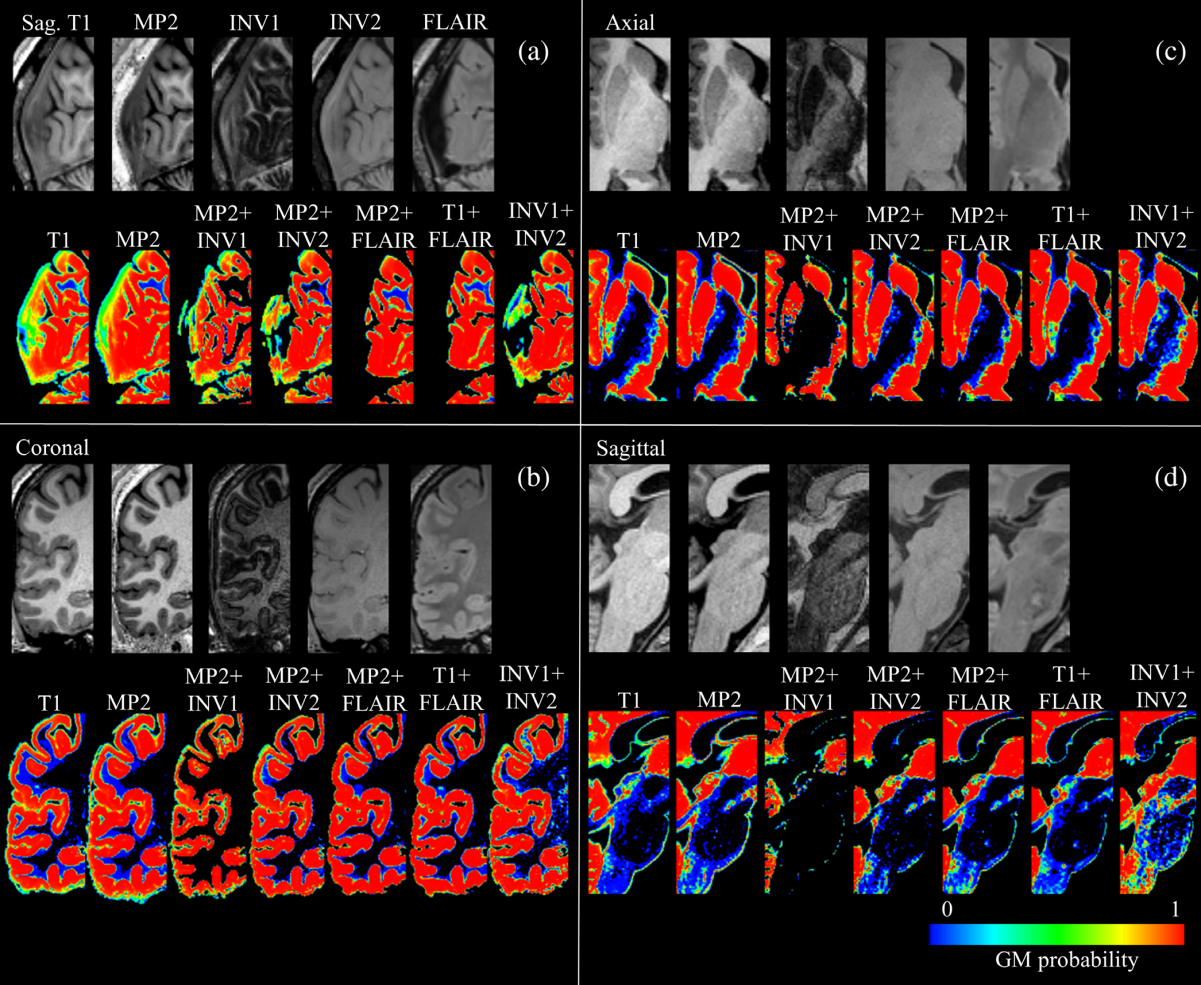
Segmentation results in the individual native space, with up‐sample from 1 to 0.5 mm^3^ for appreciating segmentation differences across all combinations. (a) Shows native T1, MP2, INV1, INV2, and FLAIR images in sagittal view, with GM segmentation probabilities (0–1) for all combinations, that is, T1, MP2, MP2 + INV1, MP2 + INV2, MP2 + FLAIR, T1 + FLAIR, and INV1 + INV2 for visual review of dura and vessels. (b) Shows the native images and segmentation results for the visual analysis of cortical segmentation, in the coronal plane. (c) Shows subcortical segmentation of thalamus and putamen (mainly posterior putamen) for all segmentation combinations in the axial view and (d) shows the segmentation of brain stem [Color figure can be viewed at http://wileyonlinelibrary.com]

We found significant differences among segmentation combinations for absolute volumes of GM (*p* < .05), WM (*p* < .05), CSF (*p* < .05), and TIV (*p* < .05; Figure 1, Data S1—Table S2). The voxel‐based group level comparisons in healthy subjects using a paired *t*‐test (*p* < .05 FWE) for different VBM combinations are presented in Figure 2 (and compare Data S1—Figures S1–S5). These results were also qualitatively confirmed through visual inspection of individual subject segmentations in native space (Figure 3, Data S1—Figures S6–S8). First, we found that the meninges and venous sinuses (straight, transverse, and sigmoid) were mostly misclassified as GM by T1, MP2, and INV1 + INV2 (Figure 3a). Additionally, for single‐contrast MP2, there was also misclassification of meninges to GM in the frontal pole, superior, middle, and inferior temporal gyrus (Figure 3b). With the addition of FLAIR, misclassifying these structures to GM was clearly reduced. Addition of INV1 and INV2 to MP2 also resolves the misclassification of meninges to GM, most apparent in the temporal and the occipital lobe (Figure 3a,b). MP2 + FLAIR/INV1/INV2 combinations segmented meninges and vessels as CSF tissue class (Data S1—Figure S8). We also observed that the cortical ribbon was segmented consistently thinner in MP2 + INV1 when compared to all other approaches (compare Figure 3b and Data S1—Figure S9). Furthermore, in comparison to T1 + FLAIR, except MP2 + INV1, all segmentations classified parts of thalamus and mainly posterior putamen as GM. Upon visual inspection, none of the models completely segmented thalamus as GM. However, better segmentation results were achieved by MP2, MP2 + INV2/FLAIR, and INV1 + INV2, also in comparison to T1 (Data S1—Figures S3–S5). Furthermore, MP2 along with its combination variants (FLAIR/INV2 and INV1 + INV2) displayed a better segmentation of posterior putamen as GM with probabilities ≥0.75–1.0 (Figure 3c). In fact, a major difference between T1 + FLAIR, MP2 + FLAIR, and MP2 + INV2 was in subcortical segmentation of parts of basal ganglia (Figure 2 and Figure 3c). Otherwise, these three combinations are mostly similar to each other. MP2 + INV1 mostly misclassified these structures as WM. Also, the rest of the models including T1 + FLAIR misclassified portions of thalamus, putamen and majority of globus pallidus as WM. Finally, we found that INV1 + INV2 yielded high GM probabilities (≥0.5–1) for the brain stem including portions of the mid brain and medulla in comparison to all segmentation approaches (Figure 2, Figure 3d, Data S1—Figure S6). Pons was partially segmented as GM by INV1 + INV2. MP2 and its combination with INV2 showed increased GM volumes in the pons (Figure 2) for group comparisons at (*p* < .05 FWE).

### 3.2 Individual VBM based on MP2 and multispectral variants in focal epilepsy

**Figure 4:**
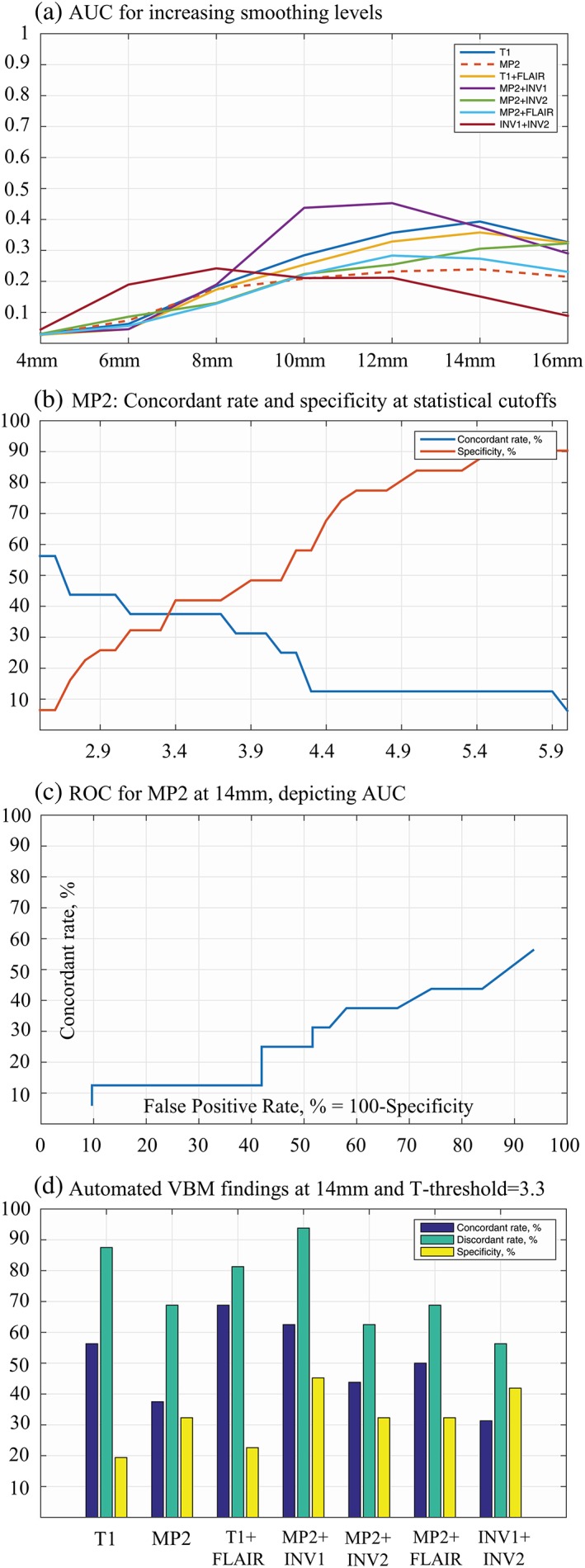
Assessment of smoothing and statistical cutoffs with MP2RAGE (MP2) as reference. Performance of different smoothing levels (a), the plot for concordant rates and specificity versus increasing statistical cutoffs (b), receiver operating characteristic (ROC) curve at 14 mm smoothing for MP2 (c), and automated VBM findings for all models at 14 mm smoothing and 3.3 T‐threshold (d) are present with this figure [Color figure can be viewed at http://wileyonlinelibrary.com]

The best performing smoothing level with single‐contrast MP2 VBM as the reference method was found at 14 mm, (AUC = 0.24, Figure 4a,c and Data S1—Table S3). At this smoothing level, T1 had the highest AUC (0.39) among all models followed by MP2 + INV1 (AUC = 0.38). For 14 mm, we found the optimal T‐threshold at 3.3 (Figure 4b).

#### 3.2.1 VBM results using the optimized parameters

While incorporating optimized VBM parameters (smoothing = 14 mm, T‐threshold = 3.3) to the lesional MRI epilepsy cohort, we found a sensitivity between 80 and 100% between the models, except in this case of INV1 + INV2 with a sensitivity of 60%. One hundred percent sensitivity across all models could be achieved at 14 mm smoothing, but with a much lower T‐threshold of at least 2.3 (Table 1).

**Table 1:** Effect sizes/T‐scores for lesional MRI patients

Model	T1	MP2	T1 + FLAIR	MP2 + INV1	MP2 + INV2	MP2 + FLAIR	INV1 + INV2
Smoothing = 14 mm, T‐threshold = 3.3							
Case 1	7.9	10.1	11.2	9.4	9.1	11.7	8.9
Case 4	2.5#	2.5#	5.3	2.3#	4.2	4.7	3.1#
Case 16	4.2	3.8	3.9	3.9	3.9	3.9	5.3
Case 17	3.6	3.3	3.2#	3.8	3.1#	3.5	2.4#
Case 21	4.6	4.8	6.8	6.9	5.7	7.5	5.9
Sensitivity (%)	80	80	80	80	80	100	60

*Note*: This table shows the maximal T‐scores (cluster maximum) for MRI‐positive (MCD) patients at a smoothing of 14 mm, for all VBM models. At a T‐threshold = 3.3, the VBM variants had a sensitivity between 60 and 100%. # indicates findings below the critical T‐threshold of 3.3.

The results of the MRI‐negative cohort are summarized in Tables 2, 3, 4. In the automated lobar‐based VBM analysis, MP2 + INV1 was the best VBM variant with ED = 66.4, concordant rate = 62.5%, specificity = 45.2% and concordant to discordant ratio = 0.67 (Figure 4d, Table 2). After visual analysis, there was a decrease in the both, concordant and discordant rates. MP2 + INV1 was still the best performing model and yielded 6.3% of potentially epileptogenic (and visible) findings and 31.3% of potentially epileptogenic (and not visible) findings in the lobe of hypothesis (Table 3). This resulted in a concordant rate of 37.5%. Whereas in the discordant lobe(s), MP2 + INV1 showed 6.3% of visible lesions and 62.5% of nonvisible lesions, with an overall discordant rate of 62.5%, constituting to a concordant to discordant ratio of 0.60.

**Table 2:** Diagnostic performance of different VBM models

Model	Concordant rate (C_R_, %)	Discordant rate (D_R_, %)	Specificity (S_P_, %)	Euclidean distance (ED)	C_R_/D_R_
Automated VBM findings					
T1	56.3	87.5	19.4	91.7	0.64
MP2	37.5	68.8	32.3	92.1	0.55
T1 + FLAIR	68.8	81.3	22.6	83.5	0.85
MP2 + INV1	62.5	93.8	45.2	66.4	0.67
MP2 + INV2	43.8	62.5	32.3	88.0	0.70
MP2 + FLAIR	50.0	68.8	32.3	84.2	0.73
INV1 + INV2	31.3	56.3	41.9	90.0	0.57
Visual interpretation					
T1	18.8	68.8	35.5	103.7	0.27
MP2	18.8	43.8	45.2	98.0	0.43
T1 + FLAIR	25.0	43.8	35.5	99.0	0.57
MP2 + INV1	37.5	62.5	51.6	79.0	0.60
MP2 + INV2	31.3	37.5	45.2	87.9	0.83
MP2 + FLAIR	18.8	31.3	35.5	103.7	0.60
INV1 + INV2	6.3	18.8	54.8	104.0	0.34

*Note*: Lobar and visually interpreted VBM results for GMC analysis are shown for a smoothing of 14 mm and T‐threshold of 3.3. The lowest Euclidean distance (ED) was found for the MP2 + INV1 combination, indicating the best trade‐off between S_P_ and C_R_.

**Table 3:** Detailed results of visual interpretation of VBM findings for MRI‐negative patients

	Concordant lobe(s)					
Model	Potentially epileptogenic (and visible, %)	Potentially epileptogenic (and not visible, %)	Potentially epileptogenic (combined, %)	Nonepileptogenic (%)	Unclear (%)	Artifact (%)
T1	6.3	12.5	18.8	0	43.8	25.0
MP2	6.3	12.5	18.8	0	25.0	0
T1 + FLAIR	6.3	18.8	25.0	6.3	43.8	6.3
MP2 + INV1	6.3	31.3	37.5	12.5	43.8	12.5
MP2 + INV2	6.3	25.0	31.3	6.3	25.0	6.3
MP2 + FLAIR	6.3	12.5	18.8	6.3	31.3	6.3
INV1 + INV2	0	6.3	6.3	0	25.0	6.3
	**Discordant lobes**					
T1	6.3	68.8	68.8	37.5	68.8	25.0
MP2	6.3	43.8	43.8	25.0	37.5	12.5
T1 + FLAIR	6.3	43.8	43.8	25.0	62.5	43.8
MP2 + INV1	6.3	62.5	62.5	50.0	81.3	43.8
MP2 + INV2	6.3	31.3	37.5	31.3	43.8	18.8
MP2 + FLAIR	6.3	31.3	31.3	25.0	50.0	31.3
INV1 + INV2	0	18.8	18.8	12.5	43.8	0

*Note*: Visual analysis results for different VBM models are present in this table for both concordant and discordant lobes. The results comprise of percentage number of potentially epileptogenic (and visible), potentially epileptogenic (and not visible), potentially epileptogenic (combined), nonepileptogenic, unclear findings and artifacts. Potentially epileptogenic (combined) refers to percentage of patients who had either potentially epileptogenic lesion (visible/nonvisible or both) overlapping with the lobe of clinical hypothesis.

**Table 4:** Detailed results of visual interpretation of VBM findings for controls

Model	Nonepileptogenic (%)	Unclear (%)	Artifact (%)	Corrected specificity (%)
T1	29.0	64.5	25.8	35.5
MP2	19.4	54.8	25.8	45.2
T1 + FLAIR	29.0	64.5	29.0	35.5
MP2 + INV1	6.5	48.4	9.7	51.6
MP2 + INV2	25.8	54.8	22.6	45.2
MP2 + FLAIR	16.1	64.5	19.4	35.5
INV1 + INV2	25.8	45.2	29.0	54.8

*Note*: Shown here are VBM results for controls with percentage of nonepileptogenic, unclear, artifacts, and corrected specificity in controls, for different VBM models. Corrected specificity refers to percentage of controls without nonepileptogenic/artifact findings. Corrected specificity can be derived as 100‐unclear findings (%).

The specificity of the automated VBM analysis was between 19.4 and 45.2%, with highest specificity for MP2 + INV1. After the visual interpretation, as expected, there was an increase in specificity with the highest for INV1 + INV2 at 54.8%. The percentage of nonvisible findings in controls were slightly lower (45.2–64.5%) than in patients (56.3–93.8%, Data S1—Table S4).

#### 3.2.2 Conventional T1 and T1 + FLAIR VBM variants (nonMP2RAGE VBM)

Both the conventional VBM variants (T1‐only and T1 + FLAIR) detected known lesions on MRI with a sensitivity of 80%. For the nonlesional cohorts, the best (automated) VBM performance was found for T1 + FLAIR, with concordant rate of 68.8% (T1: 56.3%), specificity of 22.6% (T1: 19.4%), and concordant to discordant ratio of 0.85 (T1: 0.64; Table 2). After visual inspection, T1 + FLAIR was still better than T1 with concordant rate of 25.0% (T1: 18.8%), specificity of 35.5% (T1: 35.5%), and concordant to discordant ratio of 0.57 (T1: 0.27).

## 4 DISCUSSION

In this study, we have systematically compared the MP2RAGE based segmentation combinations with more conventional segmentation approaches of T1 and T1 + FLAIR. We found that MP2 with a combination of INV1 (MP2 + INV1), showed a visually thinner cortical ribbon segmentation with a better separation between CSF‐GM and GM–WM, in comparison to other single‐contrast and multispectral combinations. MP2 + INV2 provides better subcortical segmentation of thalamus, basal ganglia and mostly prevents misclassification of vessels and dura as GM. Most important, in the MRI‐negative focal epilepsy cohort MP2 + INV1 also yielded better results with a concordant rate of 37.5%, specificity of 51.6% and concordant to discordant ratio of 0.60. In the group of visibly identifiable lesions, MP2 + INV1 also had a good sensitivity with 80%.

### 4.1 Differences among MP2 and its multispectral combinations

We found that the absolute GM volumes were significantly higher in both single‐contrast approaches: T1 (55 mL) and MP2 (161 mL) with respect to T1 + FLAIR. One important reason is the misclassification of dura and vessel sinuses as gray matter. The overestimation is mainly due to the close proximity and similarity in signal intensity of these structures to the cortex (Lindig et al., 2018; Viviani, Pracht, et al., 2017). The addition of FLAIR did resolve this misclassification for both the T1 and MP2. Combinations of MP2 with INV1 or INV2 also showed a similar effect of attenuating dura/vessels from GM. Second, we found that MP2RAGE variant MP2 + INV1 produced significantly lower GM volumes (71.9 mL) in comparison to T1 + FLAIR and all the other segmentation methods (96.1–233 mL). Upon visual inspection, we also observed a thinner GM segmentation using MP2 + INV1 for the cortical ribbon in most areas. The most likely reason for this is the “dark rim” of hypointense signal at the GM–WM junction. This “dark rim” has also been observed in the previous studies at 7 T using acquisition techniques such as null point imaging and tissue border enhancement by inversion recovery, facilitating enhanced visualization of tissue boundaries of interests (Costagli et al., 2014; Mougin et al., 2016). This effect is likely due to similar longitudinal magnetizations of GM and WM but with opposite polarities (Pitiot, Totman, & Gowland, 2007). An underlying biological reason could be the dependency of local T1 values on the density of myelin (Stuber et al., 2014). Third, in comparison to all segmentation approaches, MP2 and its combinations of MP2 + INV2 and MP2 + FLAIR, also INV1 + INV2 showed a better subcortical segmentation of thalamus and posterior putamen. This is in line with a recent study where MP2RAGE yielded greater reproducibility of subcortical GM including thalamus and putamen, when compared with T1 (Okubo et al., 2016; Streitburger et al., 2014).

### 4.2 VBM in focal epilepsy

#### 4.2.1 Smoothing and statistical cutoffs

It has been previously shown that the selection of smoothing (12 mm with T1 as reference) as well as statistical threshold (*t*‐score = 3.7) affected the detection rates in MRI‐negative cohort (Kotikalapudi et al., 2018). In the current study, a smoothing of 14 mm at a liberal T‐threshold of 3.3, gave the optimal trade‐off between specificity and concordant rate for the MP2 contrast. This is similar to our previous results, where increased smoothing level is compensated with decreased T‐score (Kotikalapudi et al., 2018). The worst performance was found at 4 mm smoothing, reflected by minimal AUC across all models. This is also line with previous studies on single patient comparisons, which shows that reducing kernel size to 4 or 8 mm reduces experimental design robustness and results are prone to more false positives (Kotikalapudi et al., 2018; Salmond et al., 2002). In Salmond et al. (2002) study, 12 mm smoothing was suggested for single patient comparisons. This is closer to our obtained smoothing of 14 mm and the difference in performance from 12 to 14 mm in our study is only 0.01 in AUC for MP2. Therefore, a smoothing of 12 mm could have also been considered, but at a higher statistical cutoff of 4.2 to yield comparable results. As a further validation step for the obtained parameters, we found the expected VBM sensitivity for patients with visible lesions within 60–100%, which is in line with previous studies based on lesional cohorts (Lindig et al., 2018; Martin et al., 2015). Furthermore, our results can provide guidance for maximizing the performance (concordant rate or specificity) of VBM models at a range of smoothing levels and statistical cutoffs.

#### 4.2.2 Visual interpretation of VBM findings

It can be expected that visual interpretation improves the specificity by eliminating false positive findings through expert knowledge. As predicted, specificity across models was higher after visual inspection, but also resulting in a decreased concordant rate, which is in line with previous studies (Kotikalapudi et al., 2018; Martin et al., 2017; Wang et al., 2015). The automated VBM process also had a relevant high number of discordant findings, which reduced after the visual analysis. However, it should be noted that clinical hypothesis was based on noninvasive data in most patients, which is limited by propagating of seizure activity (Alarcon et al., 2001; Spencer et al., 1985). Hence, the discordant findings may still hold clinical significance, though this cannot be resolved at this point. It can also be the case that some of these patients have multi‐focal lesions.

#### 4.2.3 Diagnostic significance of MP2 and multispectral MP2 combinations

**Figure 5:**
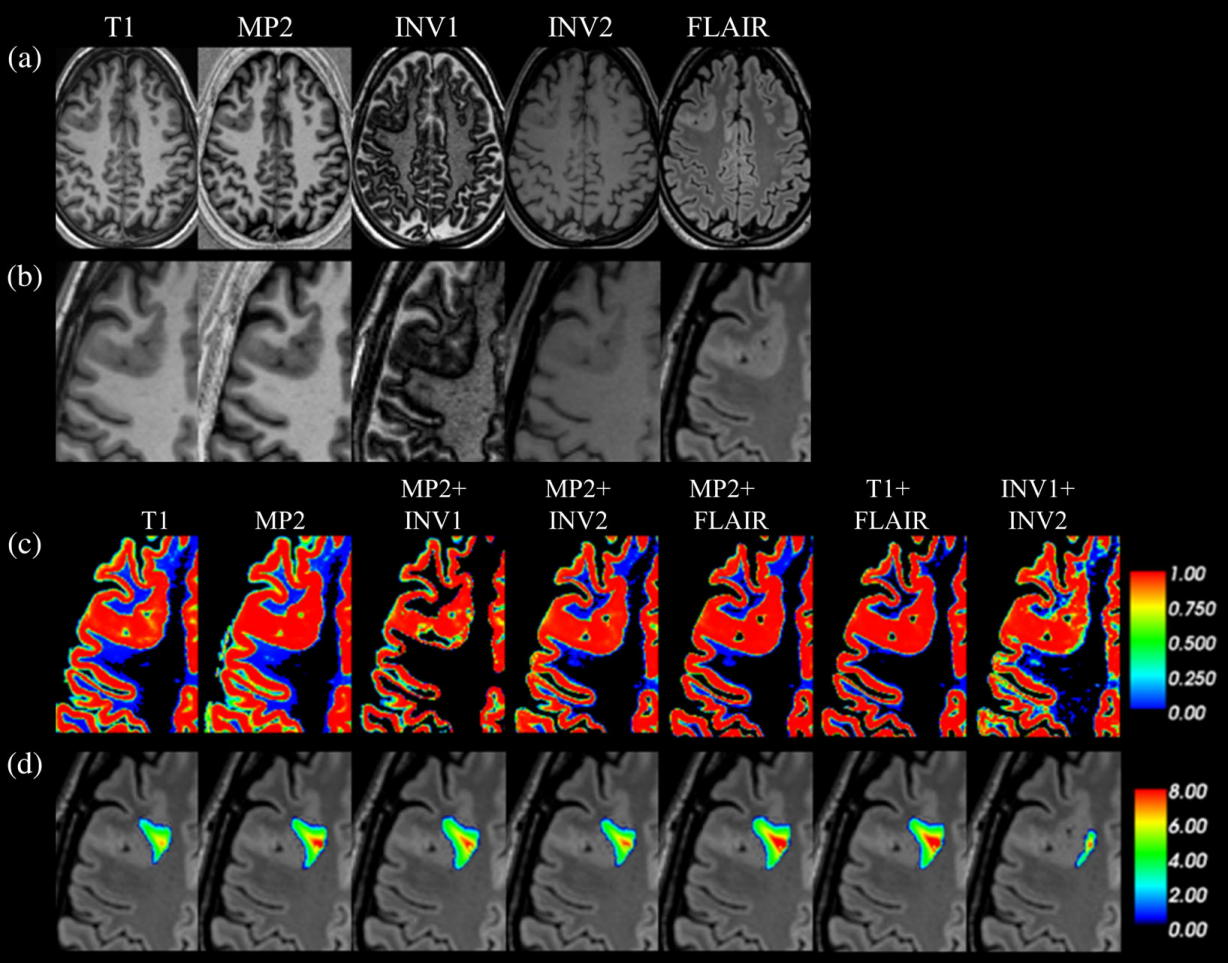
VBM results for all segmentation approaches; single‐channel and multispectral segmentations in histopathologically proven FCD type IIb. Native T1, MP2, INV1, INV2, and FLAIR images in axial view (a), zoom‐in version of the native images at the region of interest (b), segmentation approaches; T1, MP2, MP2 + INV1, MP2 + INV2, MP2 + FLAIR, T1 + FLAIR, and INV1 + INV2 for the lesion (c), VBM findings after SPM GLM models for two‐sample *t*‐test for all the VBM variants (single‐channel and multispectral), at smoothing of 14 mm and statistical cutoff of 3.3 (d). It can be observed that MP2 and MP2 based VBM variants also segment the affected area in the lesion as GM, with lesion appearing as hypo intense on all T1‐weighted images (T1, MP2, INV1, and INV2) and hyper intense on FLAIR image [Color figure can be viewed at http://wileyonlinelibrary.com]

**Figure 6:**
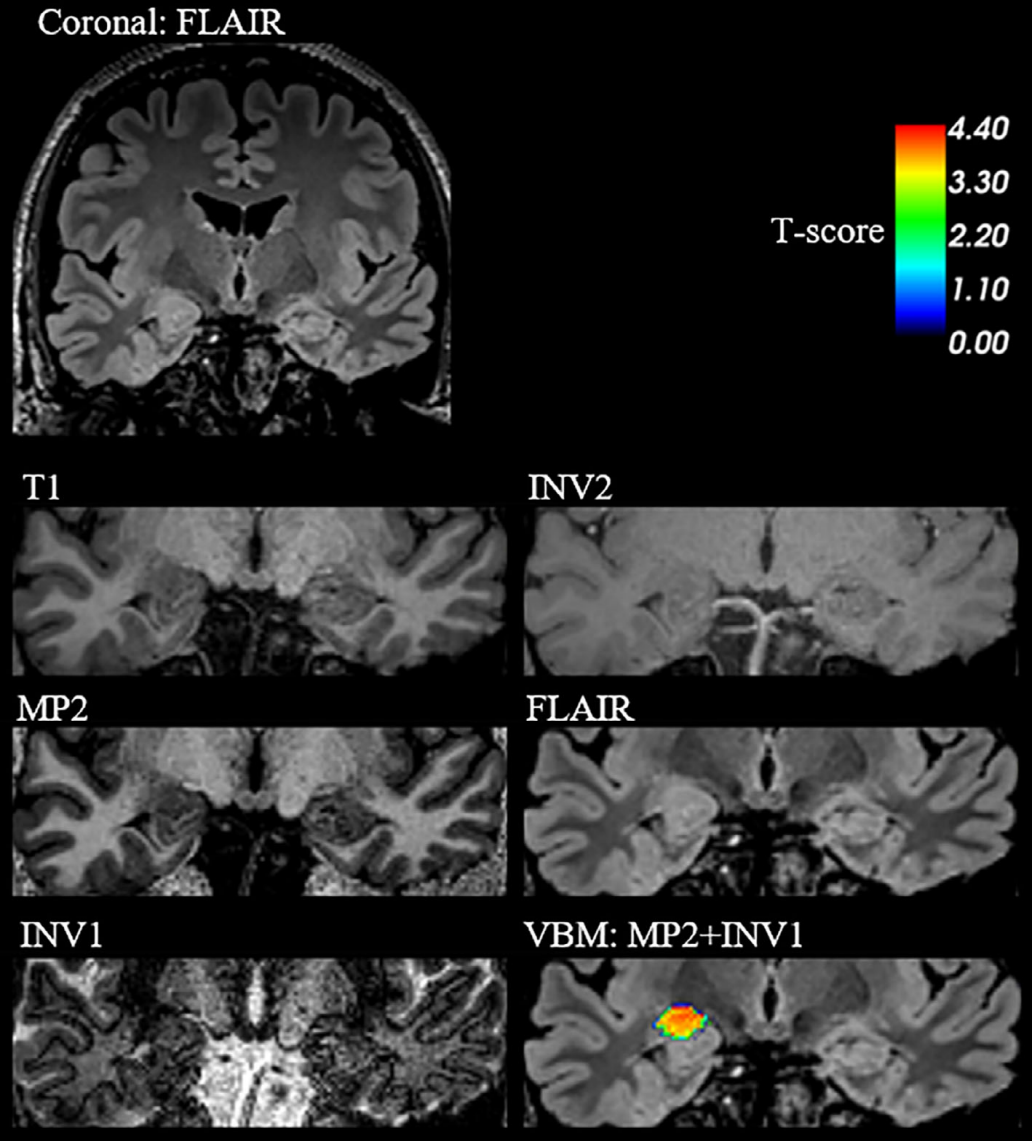
VBM results for an MRI‐negative patient detected only by MP2 VBM variant; MP2 + INV1, presented in the coronal view. Hypertrophy in the right amygdala region is most visible on FLAIR image. MP2 + INV1 VBM detects this abnormality after individual patient versus control group comparison, at smoothing of 14 mm and statistical threshold of 3.3. This finding is supported by clinical hypothesis of right temporal lobe, also supported by noninvasive EEG and neuropsychological evaluations, suspecting abnormality in the right temporal lobe [Color figure can be viewed at http://wileyonlinelibrary.com]

In a recent qualitative assessment in lesional epilepsy cases at 7 T, epileptogenic characteristics (cortical thickening, cortical–subcortical atrophy, and blurred GM–WM junction phenomena) were well appreciated on MP2RAGE images (6/7 cases = visual sensitivity 85.7%; Pittau et al., 2018). This is similar to our study, where MP2 (80%) and MP2 VBM variants showed a sensitivity between 80 and 100% in the lesional cohort (*n* cases = 5). One such example case is presented with Figure 5, for a patient with histopathologically proven FCD Type IIb in the right frontal lobe. The patient was operated and has been seizure free for the last 2.5 years. All models segment the affected area as GM, likely due to the isointensity with normal cortex. In this lesional/MRI‐positive case, MP2RAGE did not offer any substantial benefit over conventional sequences in terms of VBM lesion detection. One case from the MRI‐negative cohort is represented in Figure 6. Seizure onset was presumed in the right temporal lobe supported by noninvasive EEG recordings and neuropsychological evaluations. Though an intracranial EEG was indicated, it has not been pursued till date. In this case, MP2 + INV1 alone revealed gray matter increase in the area around the right amygdala, which is concordant to the clinical hypothesis. Improved performance by adding INV1 is in line with recent studies, which show that lesion detection could be enhanced by highlighting GM–WM boundaries (Costagli et al., 2014; Mougin et al., 2016).

### 4.3 Technical and diagnostic considerations

The approach and results demonstrated in our study should be assessed carefully, keeping in mind the technical and diagnostic considerations.

#### 4.3.1 Technical considerations

In our study, the acquisition times required for 3D T1, MP2RAGE and FLAIR at 1 mm isotropic resolution were 5:12 (minutes: seconds), 8:52 and 6:32, respectively (Data S1—Table S5). Here, the acquisition time for MP2RAGE is higher than that of T1, considering a single‐contrast VBM approach. However, acquiring MP2RAGE can be advantageous over the combination T1 + FLAIR (11:44 vs. 8:52). A better separation of tissues of interest, that is, GM–WM tissue classes, was achieved using the MP2 + INV1 multispectral segmentation. As the TI increases, the “dark rim” of hypointense voxels will shift toward the GM‐CSF (Pitiot et al., 2007). In our study, the inversion time of INV1 was 700 ms, which results in the “dark rim” appearing at the GM–WM border. For epileptogenic lesions that are often characterized by GM–WM junction smearing/expansion, such a contrast enhancement seems to aid in the lesion detection. One might also speculate that a thinner cortical segmentation might as well be sensitive toward lesions with a blurred GM–WM junction. The hypointense GM–WM boundary rim has been observed at inversion times between 200 and 1,000 ms with different sequence parameter optimizations at variable field strengths of 3 and 7 T (Costagli et al., 2014; Marques et al., 2010; Mougin et al., 2016; Pitiot et al., 2007). Hence, the selection of TI for INV1 needs to be carefully considered when planning an MP2RAGE study, as this can clearly influence the segmentation. In this study, the main focus lies in the application of MP2RAGE VBM (MP2‐only, MP2 with combination of INV1 and INV2). Thus, MP2 VBM was used as a reference model. Different reference models and cluster cutoffs could have been used and would likely yield slightly different optimized smoothing and statistical cut‐offs. Interestingly the reference model used (MP2) was not the overall best performing model. Hence, it is unlikely that this ranking of the models was strongly driven by the reference choice.

#### 4.3.2 Diagnostic considerations

From the cohort of MRI‐negative patients, no subject has yet undergone surgical resection. Hence, it is not possible to histologically confirm the VBM findings. Given the limited specificity, it is important that the automated VBM findings are validated in a careful visual interpretation to improve diagnostic relevance. In our sample, the cumulative ratio of concordant‐to‐discordant lobes was 0.27, that is, we had 27 lobes concordant to the hypothesis and 101 lobes considered as discordant in the cohort of MRI‐negative patients. With the exception of T1‐only, all the models had a concordant‐to‐discordant rate ratio of above 0.27 that would be explainable by chance. The best model in this index was MP2 + INV2 (0.83), followed by MP2 + INV1 and MP2 + FLAIR (0.60 each; compare Table 2). However, to fully understand the diagnostic yield of VBM approaches, large multicenter studies in epilepsy with seizure freedom and histopathological evidences are required. Our results could offer guidance in planning such studies while incorporating strategies to maximize VBM performance.

### 4.4 Conclusion

In this study, we have systematically compared existing single‐contrast and multispectral segmentation models T1 and T1 + FLAIR with newer models based on MP2RAGE. Further, we have compared the performance across these models for VBM in focal epilepsy patients with and without a negative conventional MRI. We found that segmentation based on MP2RAGE combinations hold different advantages for different models. A finer cortical segmentation for GM can be achieved using MP2 + INV1, while misclassification of meninges and vessels as gray matter (GM) can be addressed via MP2 + INV2. Also, improved subcortical GM classification for thalamus and posterior putamen can be achieved using MP2 + INV2. For MRI‐negative focal epilepsy, we found better performance for MP2 + INV1 in detecting GM structural abnormalities at 14 mm and statistical cutoff of 3.3 with concordant rate, specificity, and concordant to discordant ratio of 37.5%, 51.6% and 0.60, respectively. At the same smoothing kernel size and statistical cutoff, a sensitivity of 60–100% was achieved across all VBM models for known lesional MRI cases with focal epilepsy. In conclusion, we find MP2RAGE‐based multispectral VBM feasible and partially superior to the best available common VBM variants (T1 and T1 + FLAIR) in the challenging cohorts of focal epilepsy. Also, the self‐bias corrected MP2RAGE sequences hold an additional advantage to be useful at ultra‐high fields (≥7 T).

## CONFLICT OF INTEREST

The authors do not have any conflict of interest to declare.

## Supporting information


**Data S1**: Supporting Information
