# Supplementary material for: MP2RAGE multispectral voxel‐based morphometry in focal epilepsy
Source: Hum Brain Mapp. 2019 Aug 12;40(17):5042–55. doi: 10.1002/hbm.24756 (PMC6865377; doi:10.1002/hbm.24756)
Supplement: Supplementary file 1 — Data S1: Supporting Information [file HBM-40-5042-s001.docx]

Supplementary table 1: Clinical details of patients with focal epilepsy.

| **Case ID** | **Age** | **Sex** | **Onset** | **Hypothesis** | **PET – CT/MRI** | **Intracranial EEG** | **Surgery**  **(reason for deferral)** | **Surgical Outcome**  **(Engel Class,** **duration)** | **Histopathological findings** | **Neuro-psychology** | **Non-invasive Video-EEG Monitoring** |
| --- | --- | --- | --- | --- | --- | --- | --- | --- | --- | --- | --- |
| 1  (MR Visible) | 22 | f | 5 | R F | - | - | yes | I (2.5 years) | FCD IIb | F | R F |
| 2 | 28 | f | 18 | L/R T | L T | L+R T | -  (multifocal) | - | - | L F-T | L/R T |
| 3 | 19 | m | 10 | R T-P-O | normal | R T-P-O | -  (eloquent area) | - | - | R F-T | R T-P-O |
| 4  (MR Visible) | 30 | f | 9 | R I/L O | - | - | - | - | - | F | F/T |
| 5 | 19 | m | 4 | R F-T | normal | - | - | - | - | R F-T | R F-T |
| 6 | 21 | m | 14 | L T-P | normal | L T-P | -  (multifocal) | - | - | L F | L F-T-P |
| 7 | 60 | m | 20 | L/R T | - | - | - | - | - | L/R F-T | L T |
| 8 | 22 | f | 14 | L F | - | - | - | - | - | L/R F | L F |
| 9 | 21 | f | 12 | R F | - | - | - | - | - | R F-T | R F |
| 10 | 18 | f | 13 | L/R F | normal | - | - | - | - | L/R F | L/R F |
| 11 | 28 | m | 26 | R F-T | - | - | - | - | - | - | Routine-EEG R F-T |
| 12 | 27 | m | 14 | L/R T | normal | - | - | - | - | R F-T | L/R T |
| 13 | 22 | m | 16 | L/R F | normal | - | - | - | - | L/R F | L/R F |
| 14 | 33 | m | 16 | R T | normal | - | - | - | - | R F-T | R F-T |
| 15 | 26 | m | 13 | L F | normal | - | - | - | - | L/R F | L F |
| 16  (MR Visible) | 47 | m | 32 | L F | - | L F | yes | I (2 years) | FCD IIb | L F-T | L F |
| 17  (MR Visible) | 31 | f | 3 | L F | L F | L F | yes | not yet available  (3 months) | Fragmented tissue, no evidence of cortical dysplasia | L F-T | L F |
| 18 | 32 | f | 18 | L T | - | - | - | - | - | L F-T | L T |
| 19 | 51 | f | 30 | L/R T | - | - | - | - | - | L/R F-T | L/R F-T |
| 20 | 44 | m | 14 | L T | - | - | - | - | - | L F-T | L/R F-T |
| 21  (MR Visible) | 40 | f | 1 | R F | normal | R F | - | - | - | R F-T | R F |

Clinical details comprising of subject ID, age, gender, age of onset, Clinical MRI, PET-CT/MRI, clinical hypothesis, intracranial EEG, outcome of surgery, seizure freedom, histopathological findings, neuropsychological assessment and non-invasive scalp EEG information are presented with this table. This information contains both MRI-negative and MRI-visible lesional (suspected MCD) cases. L = left, R = right, T = temporal lobe, F = frontal lobe, P = parietal lobe, O = occipital lobe, I = insular lobe. ‘–’ indicates not done/not available.

Supplementary table 2: Comparison of absolute tissue volumes for different segmentation combinations.

| **GM** | **Mean(ml)±SE** | **T1** | **MP2** | **T1+FLAIR** | **MP2+INV1** | **MP2+INV2** | **MP2+FLAIR** | **INV1+INV2** |
| --- | --- | --- | --- | --- | --- | --- | --- | --- |
| **T1** | 731.5±9.7 |  | 106.1±3.2 | 55.0±3.0 | 126.9±3.7 | 23.9±3.1 | 30.8±3.4 | 32.5±3.3 |
| **MP2** | 837.6±11.3 | 106.1±3.2 |  | 161.1±4.4 | 233.0±4.2 | 130.0±4.2 | 136.9±4.8 | 73.6±3.4 |
| **T1+FLAIR** | 676.5±8.2 | 55.0±3.0 | 161.1±4.4 |  | 71.9±4.6 | 31.1±2.5 | 24.2±1.6 | 87.5±3.5 |
| **MP2+INV1** | 604.6±9.6 | 126.9±3.7 | 233.0±4.2 | 71.9±4.6 |  | 103.0±4.6 | 96.1±5.3 | 159.4±3.9 |
| **MP2+INV2** | 707.6±9.2 | 23.9±3.1 | 130.0±4.2 | 31.1±2.5 | 103.0±4.6 |  | 6.9±2.7 | 56.4±3.0 |
| **MP2+FLAIR** | 700.7±8.8 | 30.8±3.4 | 136.9±4.8 | 24.2±1.6 | 96.1±5.3 | 6.9±2.7 |  | 63.3±3.5 |
| **INV1+INV2** | 764.0±10.6 | 32.5±3.3 | 73.6±3.4 | 87.5±3.5 | 159.4±3.9 | 56.4±3.0 | 63.3±3.5 |  |
|  |  |  |  |  |  |  |  |  |
| **WM** |  |  |  |  |  |  |  |  |
| **T1** | 470.0±9.0 |  | 25.0±0.8 | 9.0±1.2 | 73.5±2.2 | 15.1±0.9 | 13.1±1.4 | 90.3±2.4 |
| **MP2** | 445.0±8.9 | 25.0±0.8 |  | 34.0±1.1 | 98.5±2.1 | 9.9±0.7 | 11.8±1.3 | 65.4±2.1 |
| **T1+FLAIR** | 479.0±9.0 | 9.0±1.2 | 34.0±1.1 |  | 64.5±2.6 | 24.1±1.1 | 22.1±0.9 | 99.3±2.8 |
| **MP2+INV1** | 543.5±10.1 | 73.5±2.2 | 98.5±2.1 | 64.5±2.6 |  | 88.6±2.4 | 86.6±2.8 | 163.8±2.7 |
| **MP2+INV2** | 455.0±8.6 | 15.1±0.9 | 9.9±0.7 | 24.1±1.1 | 88.6±2.4 |  | 1.9±1.0 | 75.3±2.2 |
| **MP2+FLAIR** | 456.9±8.6 | 13.1±1.4 | 11.8±1.3 | 22.1±0.9 | 86.6±2.8 | 1.9±1.0 |  | 77.2±2.7 |
| **INV1+INV2** | 379.7±8.0 | 90.3±2.4 | 65.4±2.1 | 99.3±2.8 | 163.8±2.7 | 75.3±2.2 | 77.2±2.7 |  |
|  |  |  |  |  |  |  |  |  |
| **CSF** |  |  |  |  |  |  |  |  |
| **T1** | 286.8±10.6 |  | 51.2±6.8 | 30.5±6.2 | 43.6±6.8 | 27.3±7.2 | 4.5±7.5 | 34.0±7.0 |
| **MP2** | 235.6±7.8 | 51.2±6.8 |  | 20.8±5.2 | 94.8±2.9 | 78.5±3.1 | 55.7±2.9 | 85.2±3.7 |
| **T1+FLAIR** | 256.4±9.3 | 30.5±6.2 | 20.8±5.2 |  | 74.1±5.1 | 57.7±5.0 | 34.9±4.9 | 64.4±5.2 |
| **MP2+INV1** | 330.4±8.5 | 43.6±6.8 | 94.8±2.9 | 74.1±5.1 |  | 16.3±3.1 | 39.1±3.4 | 9.6±3.5 |
| **MP2+INV2** | 314.1±9.3 | 27.3±7.2 | 78.5±3.1 | 57.7±5.0 | 16.3±3.1 |  | 22.8±2.1 | 6.7±2.4 |
| **MP2+FLAIR** | 291.3±9.1 | 4.5±7.5 | 55.7±2.9 | 34.9±4.9 | 39.1±3.4 | 22.8±2.1 |  | 29.5±3.0 |
| **INV1+INV2** | 320.8±10.0 | 34.0±7.0 | 85.2±3.7 | 64.4±5.2 | 9.6±3.5 | 6.7±2.4 | 29.5±3.0 |  |
|  |  |  |  |  |  |  |  |  |
| **TIV** |  |  |  |  |  |  |  |  |
| **T1** | 1488.4±22.5 |  | 29.9±7.2 | 76.5±4.8 | 9.8±6.4 | 11.7±5.8 | 39.5±6.2 | 23.9±5.8 |
| **MP2** | 1518.3±22.3 | 29.9±7.2 |  | 106.4±6.0 | 39.7±2.4 | 41.5±2.8 | 69.3±2.5 | 53.8±3.8 |
| **T1+FLAIR** | 1411.9±21.5 | 76.5±4.8 | 106.4±6.0 |  | 66.7±5.1 | 64.8±4.9 | 37.0±4.7 | 52.6±4.7 |
| **MP2+INV1** | 1478.6±21.6 | 9.8±6.4 | 39.7±2.4 | 66.7±5.1 |  | 1.9±1.5 | 29.7±1.2 | 14.1±2.9 |
| **MP2+INV2** | 1476.7±21.8 | 11.7±5.8 | 41.5±2.8 | 64.8±4.9 | 1.9±1.5 |  | 27.8±1.2 | 12.2±2.2 |
| **MP2+FLAIR** | 1448.9±21.6 | 39.5±6.2 | 69.3±2.5 | 37.0±4.7 | 29.7±1.2 | 27.8±1.2 |  | 15.6±2.4 |
| **INV1+INV2** | 1464.5±22.2 | 23.9±5.8 | 53.8±3.8 | 52.6±4.7 | 14.1±2.9 | 12.2±2.2 | 15.6±2.4 |  |

Results for repeated measures one-way ANOVA for in-between group differences in mean absolute volumes of gray matter (GM), white matter (WM), cerebrospinal fluid (CSF) and total intracranial volume (TIV) across different segmentation models. SE = standard error; shaded are significant results, p<0.05.

Supplementary table 3: **AUC at variable smoothing levels for all VBM models.**

| **Model** | **4mm** | **6mm** | **8mm** | **10mm** | **12mm** | **14mm** | **16mm** |
| --- | --- | --- | --- | --- | --- | --- | --- |
| **T1** | 0.03 | 0.06 | 0.18 | 0.28 | 0.36 | 0.39 | 0.33 |
| **MP2** | 0.03 | 0.07 | 0.18 | 0.21 | 0.23 | 0.24 | 0.21 |
| **T1+FLAIR** | 0.03 | 0.05 | 0.17 | 0.25 | 0.33 | 0.36 | 0.32 |
| **MP2+INV1** | 0.03 | 0.05 | 0.19 | 0.44 | 0.45 | 0.38 | 0.29 |
| **MP2+INV2** | 0.03 | 0.09 | 0.13 | 0.22 | 0.25 | 0.31 | 0.32 |
| **MP2+FLAIR** | 0.03 | 0.06 | 0.13 | 0.22 | 0.28 | 0.27 | 0.23 |
| **INV1+INV2** | 0.04 | 0.19 | 0.24 | 0.21 | 0.21 | 0.15 | 0.09 |

Area under curve (AUC) for all VBM models across variable smoothing levels is present in this table. AUC for each smoothing (4mm to 16mm, step size = 2) was derived from specificity and concordant rate across all statistical cutoffs (2.5 to 6, step size = 0.1).

Supplementary table 4: **Distribution of non-visible findings in controls and patients after visual analysis.**

| **Model** | **Frequency of non-visible**  **findings in controls (%)** | **Frequency of non-visible**  **findings in patients (%)** |
| --- | --- | --- |
| **T1** | 64.5 | 87.5 |
| **MP2** | 54.8 | 68.8 |
| **T1+FLAIR** | 64.5 | 87.5 |
| **MP2+INV1** | 48.4 | 93.8 |
| **MP2+INV2** | 54.8 | 68.8 |
| **MP2+FLAIR** | 64.5 | 56.3 |
| **INV1+INV2** | 45.2 | 56.3 |

Shown in this table are percentage of controls and patients with non-visible findings. In patients, non-visible findings refer to ‘epileptogenic findings which were non-visible’ and/or ‘unclear findings’. Note that patients had more non-visible findings in most models, MP2+INV1 in particular (48.4% vs. 93.8%).

Supplementary table 5: **Total scan acquisition times for different sequences in VBM variants.**

| **VBM variants** | **Acquisition times**  **(min. sec.)** |
| --- | --- |
| **T1** | 5.12 |
| **MP2** | 8.52 |
| **T1+FLAIR** | 6.32+5.12 = 11.44 |
| **MP2+INV1** | 8.52 |
| **MP2+INV2** | 8.52 |
| **MP2+FLAIR** | 8.52+6.32 = 14.84 |
| **INV1+INV2** | 8.52 |

Supplementary figure 1: **Group level differences for white matter in MP2RAGE and T1/FLAIR segmentations.**


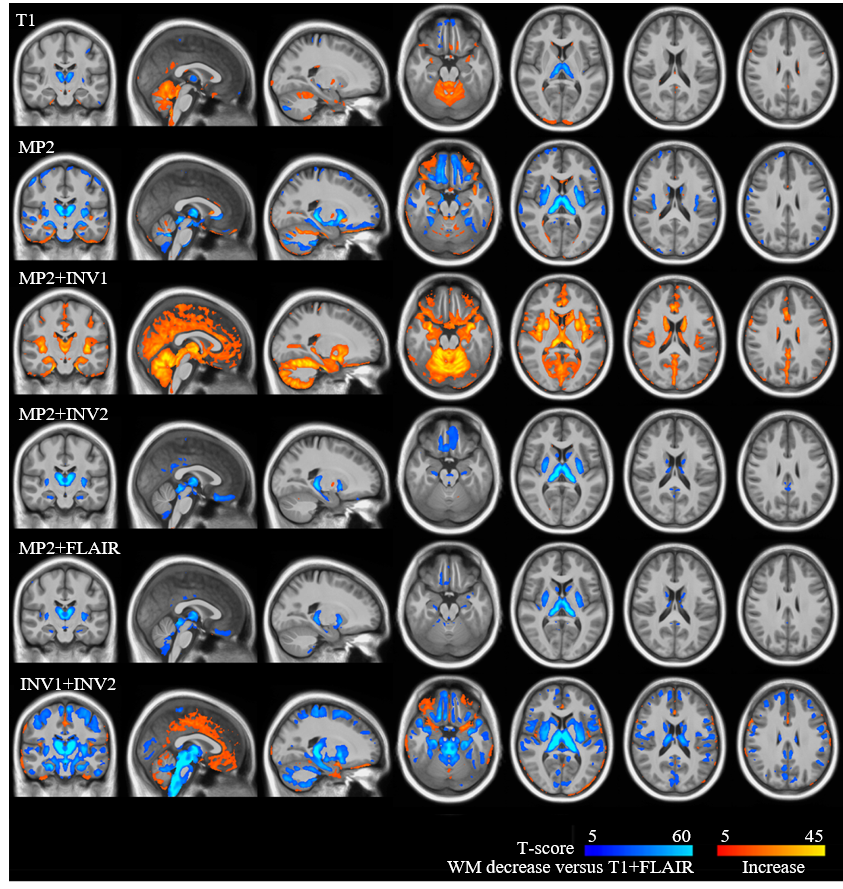


Group level comparison for T1+FLAIR with rest of the models is shown in this figure. The look-up table with red-yellow represents increased WM volumes in the compared model against T1+FLAIR (model > T1+FLAIR), while blue-light blue represents decreased WM volumes (model < T1+FLAIR). The findings are overlaid on a mean template derived from all control datasets for T1 image contrast.

Supplementary figure 2: **Group level differences for cerebrospinal fluid in MP2RAGE and T1/FLAIR segmentations.**


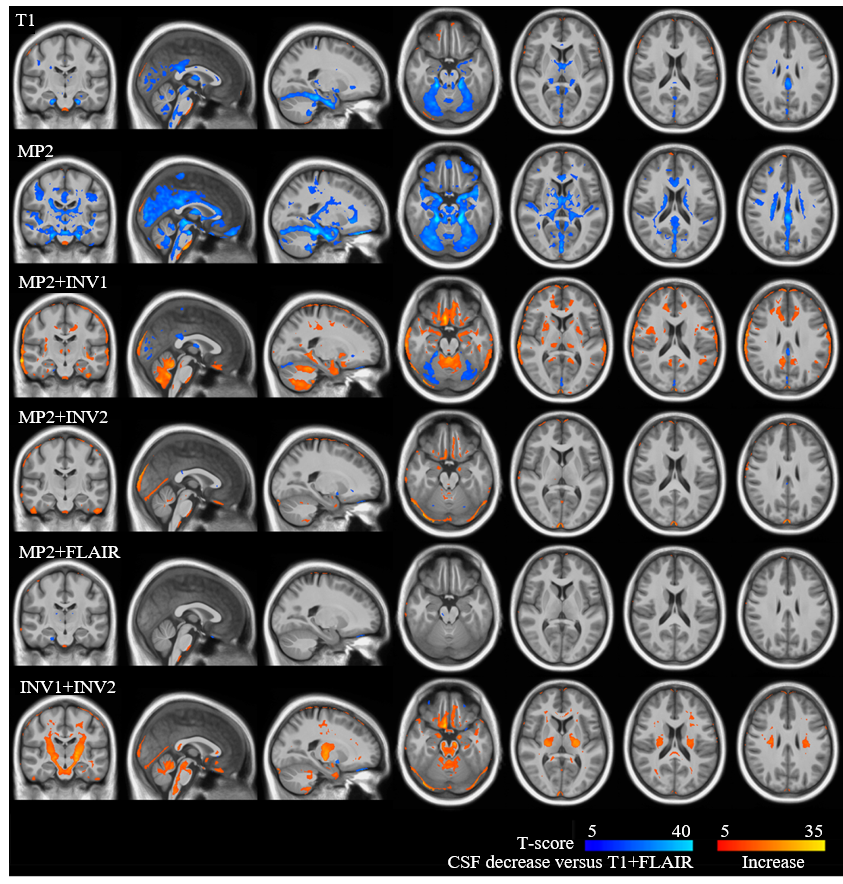


Group level comparison for T1+FLAIR with rest of the models is shown in this figure. The look-up table with Red-Yellow represents increased CSF volumes in the compared model against T1+FLAIR (model > T1+FLAIR), while Blue-light blue represents decreased CSF volumes (model < T1+FLAIR). The findings are overlaid on a mean template derived from all control datasets for T1 image contrast.

Supplementary figure 3: **Group level differences for gray matter in MP2RAGE and T1/FLAIR segmentations in comparison to T1.**


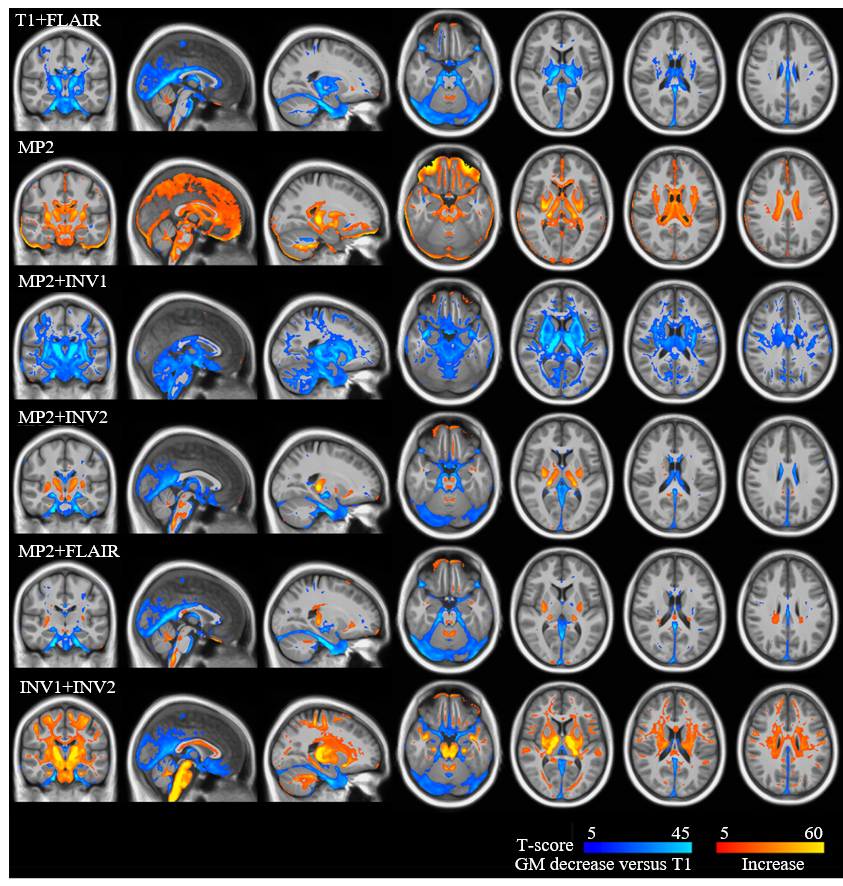


Group level comparison for T1 with rest of the models is shown in this figure. The look-up table with Red-Yellow represents increased GM volumes in the compared model against T1 (model > T1), while Blue-light blue represents decreased GM volumes (model < T1). The findings are overlaid on a mean template derived from all control datasets for T1 image contrast.

Supplementary figure 4: **Group level differences for white matter in MP2RAGE and T1/FLAIR segmentations in comparison to T1.**


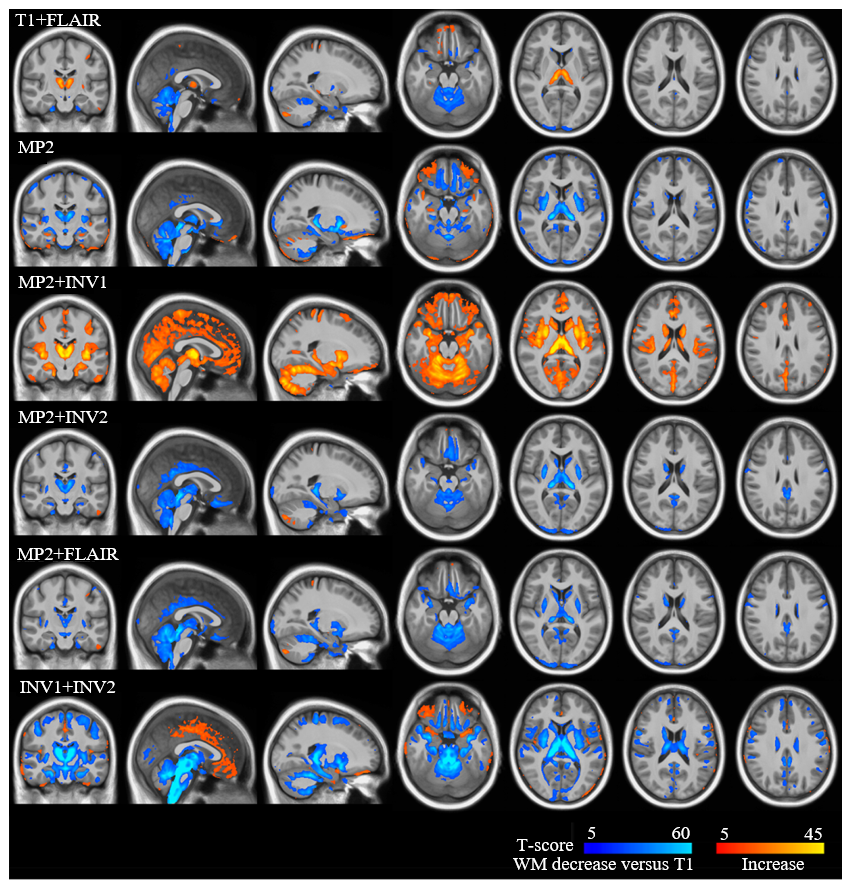


Group level comparison for T1 with rest of the models is shown in this figure. The look-up table with Red-Yellow represents increased WM volumes in the compared model against T1 (model > T1), while Blue-light blue represents decreased WM volumes (model < T1). The findings are overlaid on a mean template derived from all control datasets for T1 image contrast.

Supplementary figure 5: **Group level differences for cerebrospinal fluid in MP2RAGE and T1/FLAIR segmentations in comparison to T1.**


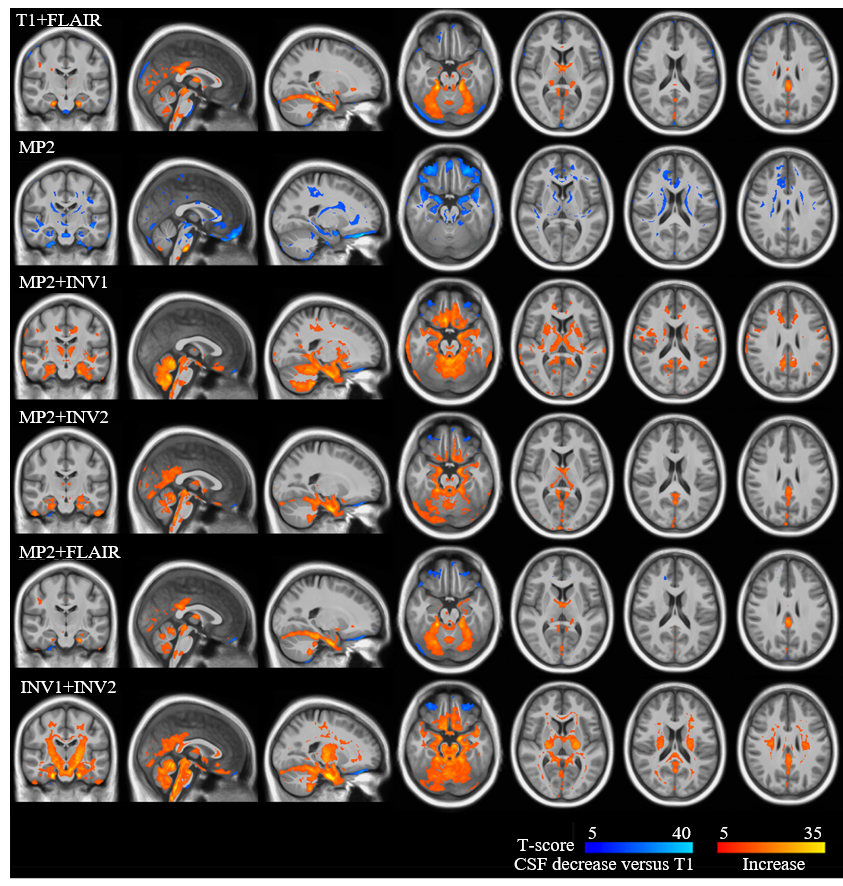


Group level comparison for T1 with rest of the models is shown in this figure. The look-up table with Red-Yellow represents increased CSF volumes in the compared model against T1 (model > T1), while Blue-light blue represents decreased CSF volumes (model < T1). The findings are overlaid on a mean template derived from all control datasets for T1 image contrast.

Supplementary figure 6: **Native space segmentation of gray matter**.


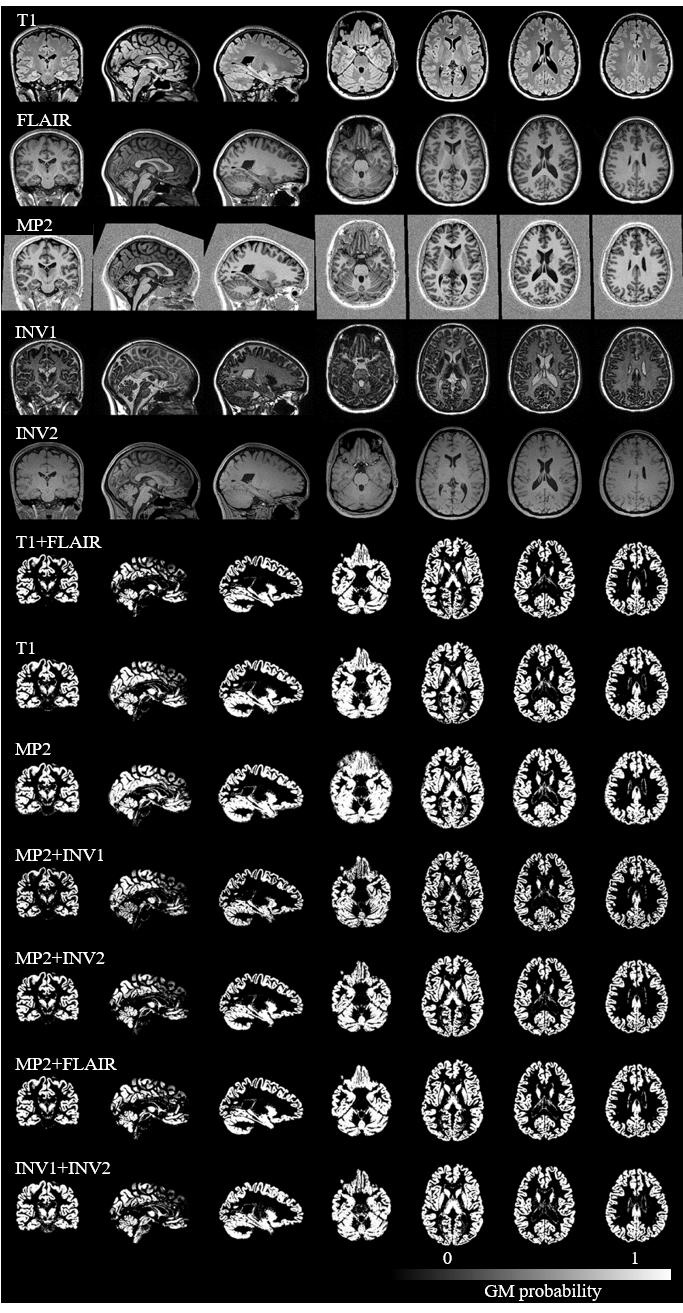


Images of GM segmentation for T1+FLAIR, T1, MP2, MP2+INV1, MP2+INV2, MP2+FLAIR and INV1+INV2 for a single control in native space are shown. Also present are the native space images of FLAIR, T1, MP2, INV1 and INV2. Images shown here and subsequent supplementary figures 7 and 8 are from the same healthy control in native space, to facilitate a systematic visual analysis.

Supplementary figure 7: **Native space segmentation of white matter.**


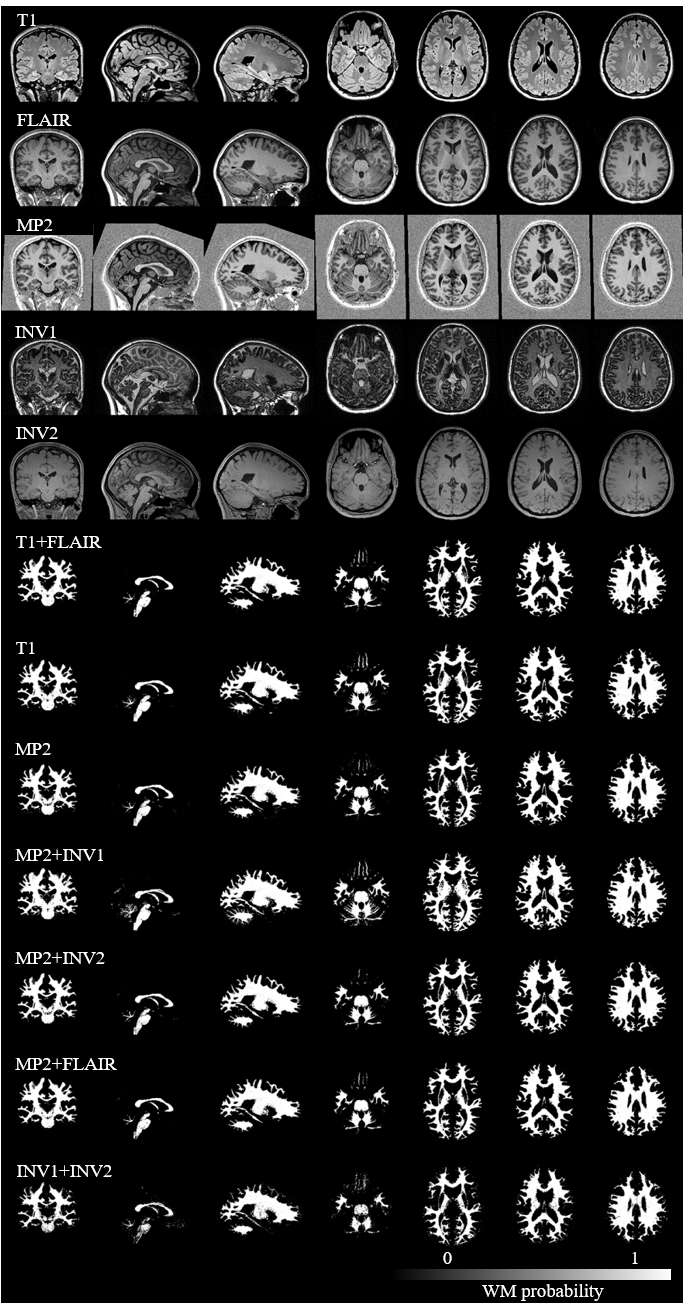


Images of WM segmentation maps for T1+FLAIR, T1, MP2, MP2+INV1, MP2+INV2, MP2+FLAIR and INV1+INV2 for a single control in native space are shown. Also present are the native space images of FLAIR, T1, MP2, INV1 and INV2.

Supplementary figure 8: **Native space segmentation of cerebrospinal fluid.**


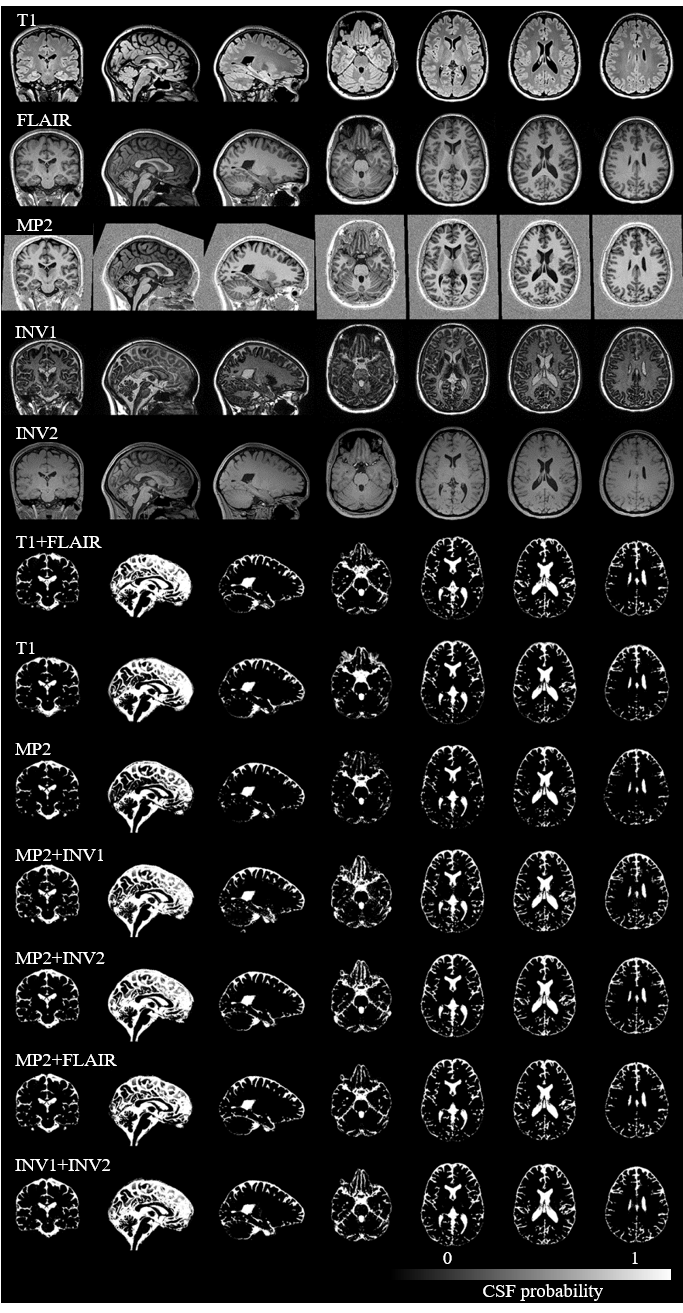


Images of CSF segmentation maps for T1+FLAIR, T1, MP2, MP2+INV1, MP2+INV2, MP2+FLAIR and INV1+INV2 for a single control in native space are shown. Also present are the native space images of FLAIR, T1, MP2, INV1 and INV2.

Supplementary figure 9: **Segmentation contours**


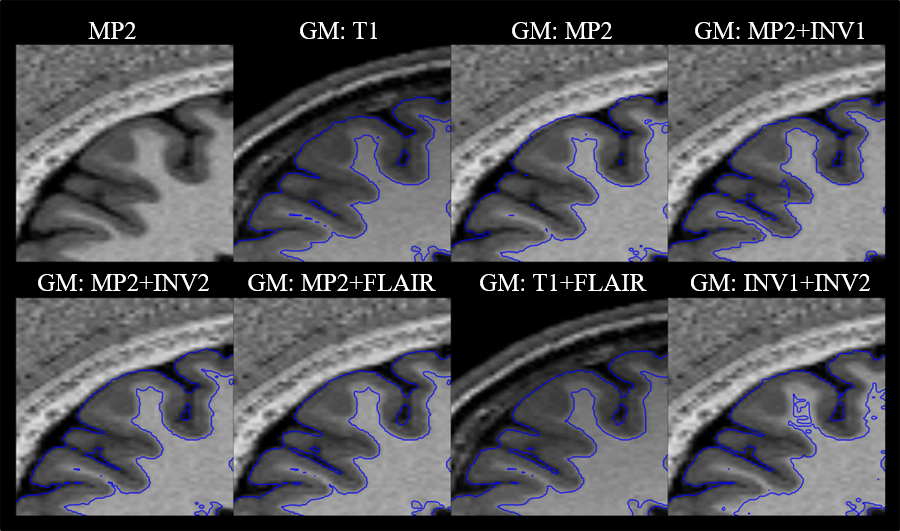


This plot shows the contours of the different GM segmentation maps. Note that the GM contours are more representative of the grey/white signal for MP2+INV1 in comparison to all the other single-channel or multi-spectral combinations. The contours are formed by voxels with a tissue probability > 0.1.

Supplementary figure 10: **Concordant rate and specificity with changing smoothing levels across statistical cutoffs**.


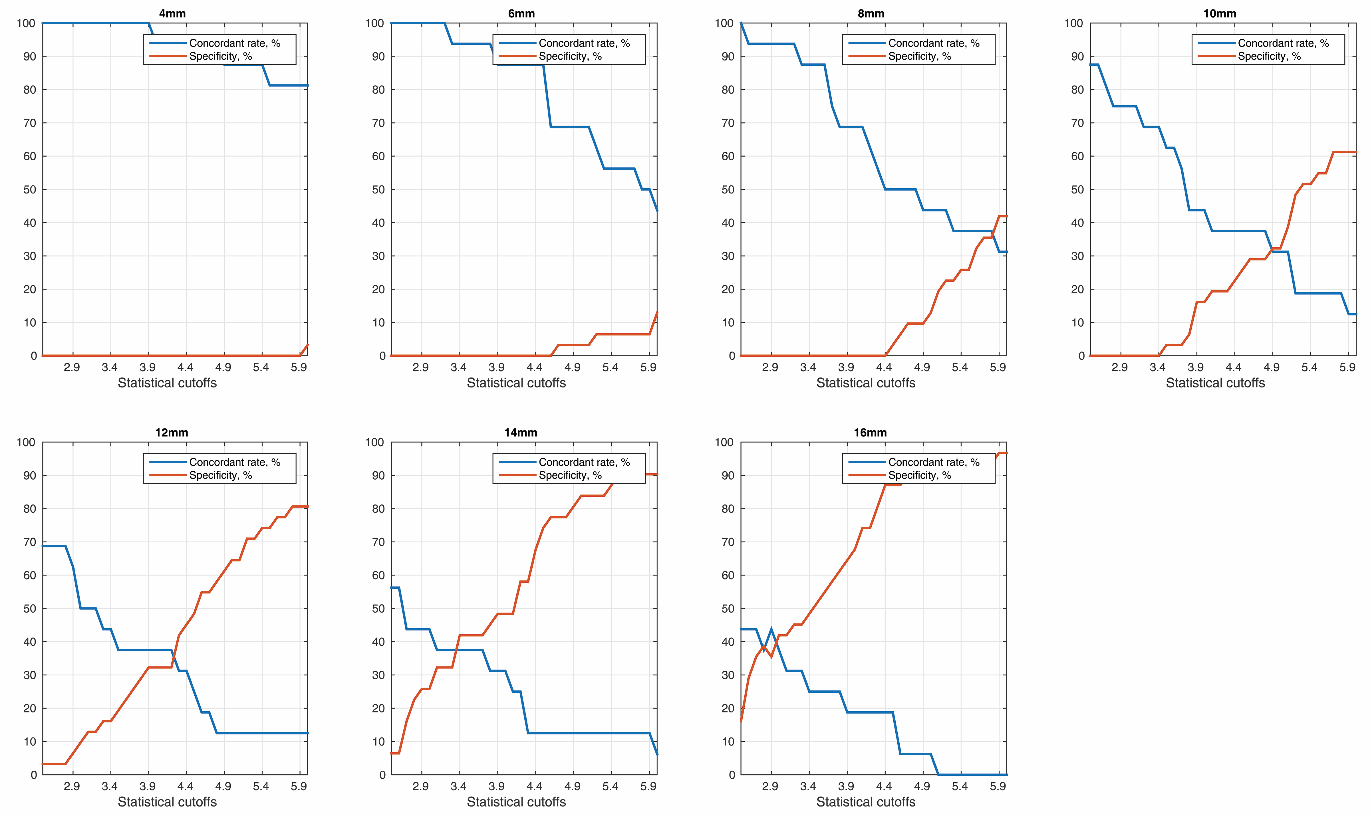


Present here are the intersection plots for concordant rates and specificity versus statistical cutoffs (T score) from 2.5 to 6 and smoothing levels (FWHM) from 4mm to 16mm for automated MP2 VBM findings. It can be observed that as the smoothing increases, the T-threshold (intersection of concordant rate and specificity curves) shifts towards the left.
